# The Assessment of the Quality of Reporting of Systematic Reviews/Meta-Analyses in Diagnostic Tests Published by Authors in China

**DOI:** 10.1371/journal.pone.0085908

**Published:** 2014-01-21

**Authors:** Long Ge, Jian-cheng Wang, Jin-long Li, Li Liang, Ni An, Xin-tong Shi, Yin-chun Liu, Jin-hui Tian

**Affiliations:** 1 Evidence-Based Medicine Center, School of Basic Medical Sciences, Lanzhou University, Lanzhou, Gansu, China; 2 The First Clinical Medicine College, Lanzhou University, Lanzhou, Gansu, China; McGill University, Canada

## Abstract

**Background:**

The quality of reporting in systematic reviews (SRs)/meta-analyses (MAs) of diagnostic tests published by authors in China has not been evaluated. The aims of present study are to evaluate the quality of reporting in diagnostic SRs/MAs using the PRISMA statement and determine the changes in the quality of reporting over time.

**Methods:**

According to the inclusion and exclusion criteria, we searched five databases including Chinese Biomedical Literature Database, PubMed, EMBASE, the Cochrane Library, and Web of knowledge, to identify SRs/MAs on diagnostic tests. The searches were conducted on July 14, 2012 and the cut off for inclusion of the SRs/MAs was December 31^st^ 2011. The PRISMA statement was used to assess the quality of reporting. Analysis was performed using Excel 2003, RevMan 5.

**Results:**

A total of 312 studies were included. Fifteen diseases systems were covered. According to the PRISMA checklist, there had been serious reporting flaws in following items: structured summary (item 2, 22.4%), objectives (item 4, 18.9%), protocol and registration (item 5, 2.6%), risk of bias across studies (item 15, 26.3%), funding (item 27, 28.8%). The subgroup analysis showed that there had been some statistically significant improvement in total compliance for 9 PRISMA items after the PRISMA was released, 6 items were statistically improved regarding funded articles, 3 items were statistically improved for CSCD articles, and there was a statistically significant increase in the proportion of reviews reporting on 22 items for SCI articles (P<0.050).

**Conclusion:**

The numbers of diagnostic SRs/MAs is increasing annually. The quality of reporting has measurably been improved over the previous years. Unfortunately, there are still many deficiencies in the reporting including protocol and registration, search, risk of bias across studies, and funding. Future Chinese reviewers should address issues on these aspects.

## Introduction

Systematic reviews (SRs) and meta-analyses (MAs) of diagnostic tests will potentially have an increasing role in healthcare as decision makers check the evidence before implementing new diagnostic technologies [1]. However, only high quality diagnostic SRs can provide the best evidence for clinical decision makers. Low quality SRs may mislead clinical practice, which may arise at a primary study level, due to flaws in the design, execution and reporting of the component studies [2], [3]. Like all other types of SRs, diagnostic SRs are also prone to a number of shortcomings [1].

In order to improve the quality of diagnostic SRs, quality evaluation of the primary studies has become an important element of the review process. The publication of the Quality Assessment of Diagnostic Accuracy Studies (QUADAS) tool [4] provides investigators with a means of assessing the major domains that affect the validity of diagnostic study. As a matter of fact, some experiences, reports, feedbacks from users or academic organizations have suggested the potential to improve this tool. Therefore, the QUADAS Group revised it and then launched QUADAS-2 [5]in 2011 [6]. The Standards for the Reporting of Diagnostic accuracy studies (STARD) initiative [3] has given investigators a reporting framework that undoubtedly improves the quality of primary studies.

SRs of diagnostic tests have been developing for nearly two decades abroad [1]. The first SR of diagnostic tests was published in Chinese journal in 2001 [7]. Since then, an increasing numbers of SRs of diagnostic tests have been published in China, however, the methodological and reporting quality of these reviews varies widely. Therefore, it is vital to assess the quality of diagnostic SRs before being used for healthcare policy or clinical decision making.

Methodological quality considers how well the SR is conducted (including literature searching, pooling of data, etc.). Reporting quality considers how well systematic reviewers have reported their methodology and findings [8]. There is no specialized tool to evaluate the quality of SRs of diagnostic research. As a newer standard of reporting SR, the Preferred Reporting Items for Systematic Reviews and Meta-Analyses (PRISMA) was released to replace the Quality of Reporting of Meta-analyses (QUOROM) for guiding the review reporting [9]. The updated PRISMA statement was based on the conceptual and practical advances that had been made in the science of SRs. The complete PRISMA statement consists of a 27-item checklist, along with a flow diagram [10]. Most of the checklist items are relevant when reporting SRs of non-randomized studies assessing the benefits and drawbacks of interventions, however, the need to modify or incorporate additional items for diagnostic accuracy studies should be recognized [11].

Willis BH et al. have investigated the quality of reporting of MAs in diagnostic researches published in English prior to December 31^st^, 2008, and found that the quality of reporting had many flaws [1]. It is unclear whether the quality of reporting of SRs/MAs on diagnostic researches published by authors in China have a similar result, and further validation is necessary. Thus, the objective of this study is to examine the quality of reporting of published SRs/MAs in diagnostic tests by authors in China, according to their compliance with the PRISMA statement.

## Methods

### Data Sources and Searches

Two independent reviewers systematically searched the following electronic databases: Chinese Biomedical Literature Database, PubMed, EMBASE, the Cochrane Library, and Web of science. Searches were conducted using a combination of the following terms: “systematic reviews”, “systematic review”, “meta analysis”, “meta analyses”, “meta-analysis”, “meta-analyses”, “sensitivity”, “specificity”, “China”, “Chinese”. The searches were implemented on July 14, 2012 and the cut off for inclusion of the SRs/MAs was December 31^st^ 2011. The syntaxes were adjusted corresponding to different database. The detailed search algorithms for each database were listed in Text S1.

### Selection

Two independent reviewers selected articles according to the inclusion and exclusion criteria designed in advance. Disagreements were resolved in consultation with Dr. Tian. After discarding duplication studies by Endnote X3 (The Thomson Reuters, Britain), we reviewed all the abstracts, identified potentially eligible articles and citations for which a decision could not be made from the abstract. We then managed to retrieve the full - text of these articles to determine whether they were eligible.

The inclusion criteria were as follows: (a) The SRs/MAs on diagnostic tests were published by authors in China. The diagnostic test was defined as a technology to distinguish between patients with disease (or more generally, a specified target disorder) and those without disease. Target disorders were considered to be pathological processes and not related to a success or failure of an intervention, such as successful placing of stents. In such a test accuracy study, the results of ‘index test’ were compared with those of the reference standard determined in the same patient. The reference standard should be the best available method for identifying patients that have the target disorders. (b) One of the objectives of the included studies was to estimate a measure of the performance of the diagnostic test; articles that reported relevant data would be extracted; (c) The qualitative and quantitative comprehensive analyses were performed for all included studies; (d) The search terms should be explicitly stated and include sensitivity or specificity.

The exclusion criteria were as follows: (a) Repeated literature, review and methodological literature, original research, animal research and case reports. (b) Conference abstracts and letters to the journal editors; (c) The SRs/MAs on other fields.

### Quality Assessment

The PRISMA statement [10] which consists of a twenty seven-point checklist was used to evaluate the overall quality of reporting of meta analyses. To indicate the degree of compliance, each checklist item was assigned one of three responses: ‘Yes’ for total compliance; ‘partial’ for partial compliance; and ‘No’ for non-compliance.

### Data Collection and Analysis

Data collection was carried out independently by two reviewers using standard data extraction forms (Table 1). All disagreements were resolved by discussion or the third researcher.

**Table 1:** Data extraction items of included studies.

	Items	Interpretation	
**Basic information**	1. Publication year	Year of publication of SRs/Mas	
	2. Source	Journal or degree paper	
	3. Language of publication	Chinese or English	
	4. Number of authors	No. authors of writing SR/MAs	
	5. Departement of authors	Number and role of authors’ departement	
	6. Gold standard	Reference standard on diagnostic tests	
	7. Index test	Evaluated tests	
	8. Information of included RCT	Language and number of included RCT	
	9. Title	Identify the report as a SR, MA, or other	
	10. Foundation item	Number and nature on foundation	
	11. Categories of disease	Condition focused on in review (ICD-10)	
	12. Quality assessment on RCT	Information of quality assessment tool on RCT	
**Quality assessment of reporting** **information**	13. Title		
	14. Abstract		
	15. Introduction		
	16. Methods	Yes/Partial/No for per item	
	17. Results		
	18. Discussion		
	19. Funding		

(Notes: RCT: Randomized Controlled Trials; SR: Systematic review; MA: Meta-analysis).

Extraction of data included the following items: title; publication year; publication journals; publication language; number of authors; affiliations of authors; number of affiliations; index test; reference standard; funding sources; categories of disease; method used to assess the quality for original studies including QUADAS, and responses to the PRISMA statement.

According to An N et al. [12], we performed subgroup analysis on the quality of reporting for total compliance in each items of PRISMA by t-test. The factors of subgroup analysis were presented as following: the year of publication (≤2008 vs. ≥2009), the affiliations (hospital vs. university), funding sources (funded vs. non-funded), Chinese Science Citation Database (CSCD) vs. non-CSCD, Science Citation Index (SCI) vs. non-SCI. The odds ratio (OR) value and 95% CI was used as the summary statistic for subgroup comparisons through Mantel-Haenszel and Std, by using a fixed-effect model. If the OR was undefined, then Fisher’s exact test was used. The ORs for each item represent responses of “Yes” for group 1 versus responses of “Yes” for group 2; OR>1 represents that the quality of group 1 is better than group 2. Statistical significance was set at P≤0.050. Analysis was performed using Excel 2003 and RevMan 5.0 software.

## Results

### Search

**Figure 1:**
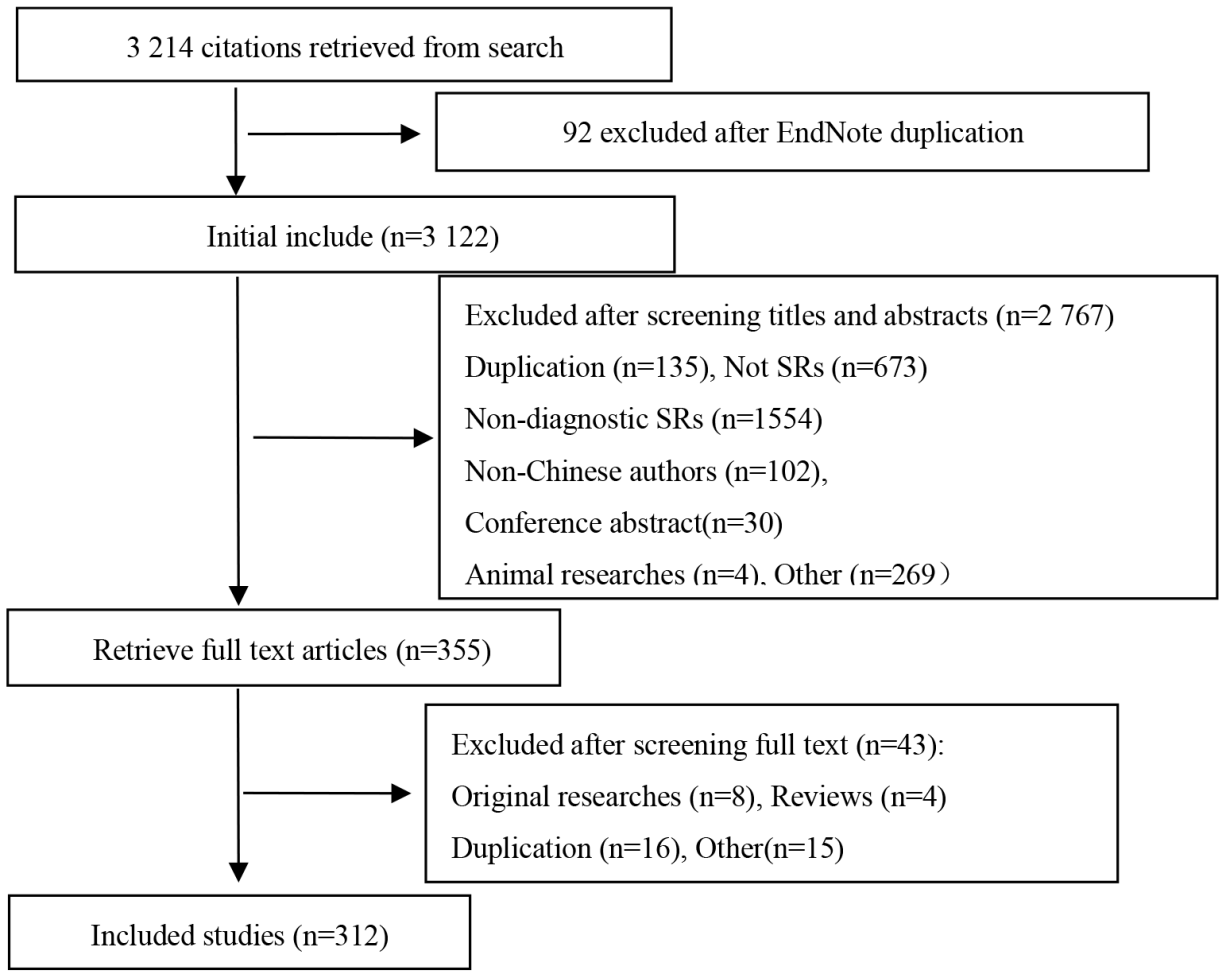
Flow chart of article screening and selection process.

3,214 records were retrieved as the result of searches. 2,767 articles were excluded due to duplication, non diagnostic tests, conference abstracts, animal researches, non Chinese authors, or not being a SR/MA. After examination of the full texts of 355 articles, a further 43 reviews were excluded because they were original researches, reviews, or duplication. A total of 312 SRs and MAs were included, 235 in Chinese and 77 in English (Figure 1).

### Characteristics of the Included SRs/MAs (Table 2)

**Figure 2:**
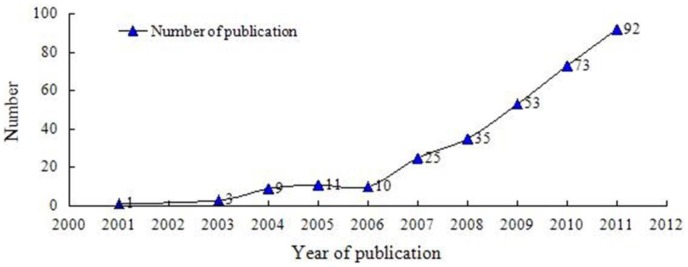
Number of included systematic reviews/Meta-analyses per year of publication.

312 SRs/MAs were written by authors in China and published in 169 different journals. Only 134 (43.0%) were published in the journals cited by Chinese Science Citation Database, with 74 (23.7%) being published in Science Citation Index Journals. In which, the impact factor (2011) was between 0.9 and 11.7, and the impact factor for 12 reviews was higher than 5.0, dispersed throughout eight journals as following: Hepatology (impact factor (IF): 11.7), Archives of Internal Medicine (IF:11.5), Thorax (IF: 6.8), Radiotherapy and Oncology (IF: 5.6), European Journal of Cancer (IF: 5.5), American Journal of Kidney Diseases (IF: 5.4), Intensive care medicine (IF:5.4), CHEST (IF:5.3). Figure 2 illustrated the number of SRs/MAs per publication year in the included set. The first diagnostic SR was published in 2001, nearly 89.0% (278 reviews) were published after the year 2007. According to ICD-10 [13], 312 studies involved 15 system diseases. The most common disorders were neoplasms (42.3%), diseases of the digestive system (10.3%), and certain infectious and parasitic diseases (10.3%). More than half of (66.4%) the SRs/MAs were written by clinicians. A wide range of diagnostic tests were featured in the reviews, with laboratory technologies (51.0%) and imaging technologies (45.2%) being the most common category of tests evaluated. 28.9% (90 reviews) reported funding sources. 88.1% (275 reviews) studies were composed by more than three authors. None of the SRs/MAs had been updated from a previous review. Characteristics of the included reviews were shown in Table 2.

**Table 2:** Characteristics of included systematic reviews/meta-analyses.

	Items	n(%)	95%CI
**Language of publications**	Chinese	235(75.3)	(70.2–79.8)
	English	77(24.7)	(20.2–29.8)
**Category of journals**	CSCD	134(57.0)	(50.6–63.2)
	Non-CSCD	101(43.0)	(36.8–49.4)
	SCI	74(23.7)	(19.3–28.8)
	Non-SCI	238(76.3)	(71.2–80.7)
**Number of authors**	≤2	37(11.9)	(8.7–15.9)
	≥3	275(88.1)	(84.1–91.3)
**Affiliations**	University	105(33.7)	(28.6–39.1)
	Hospital	207(66.3)	(60.9–71.4)
**Number of affiliations**	1	162(51.9)	(46.4–57.4)
	≥2	150(48.1)	(42.6–53.6)
**Foundation item**	Government	90(28.8)	(24.1–34.1)
	Unreported	222(71.2)	(65.9–75.9)
**Index test**	Imaging technologies	141(45.2)	(39.8–50.8)
	Laboratory technologies	159(51.0)	(45.4–56.5)
	Pathological technologies	2(0.6)	(0.2–2.5)
	Others	10(3.2)	(1.7–5.9)
**Number of included RCT**	1–5	21(6.7)	(4.4–10.1)
	6–10	88(28.2)	(23.5–33.5)
	11–15	80(25.6)	(21.1–30.8)
	16–20	43(13.8)	(14–18.1)
	≥21	78(25.0)	(20.5–30.1)
	Unreported	2(0.6)	(0.2–2.5)
**Categories of disease**	Neoplasms	132(42.3)	(36.9–47.9)
	Diseases of the digestive system	32(10.3)	(7.3–14.1)
	Diseases of the respiratory system	24(7.7)	(5.2–11.2)
	Diseases of the circulatory system	26(8.3)	(5.7–12.0)
	Certain infectious and parasitic diseases	32(10.3)	(7.3–14.1)
	Diseases of the blood and blood -forming organs	8(2.6)	(1.3–5.0)
	Endocrine,nutritional and metabolic diseases	9(2.9)	(1.5–5.4)
	Diseases of the musculoskeletal system and connective tissue	17(5.4)	(3.4–8.6)
	Diseases of the eye and adnexa	2(0.6)	(0.2–2.5)
	Diseases of the genitourinary system	10(3.2)	(1.7–5.9)
	Pregnancy, childbirth and the puerperium	3(1.0)	(0.3–2.9)
	Congenital malformations, deformations andchromosomal abnormalities	7(2.2)	(1.1–4.6)
	Symptoms, signs and abnormal clinical and laboratory findings,not elsewhere classified	2(0.6)	(0.2–2.5)
	Injury, poisoning and certain other consequences of external causes	5(1.6)	(0.7–3.8)
	Certain conditions originating in the perinatal period	3(1.0)	(0.3–2.9)

(Notes: RCT: Randomized Controlled Trials; CSCD: Chinese Science Citation Database; SCI: Science Citation Index).

### Tools of Quality Assessment of Primary Studies

**Figure 3:**
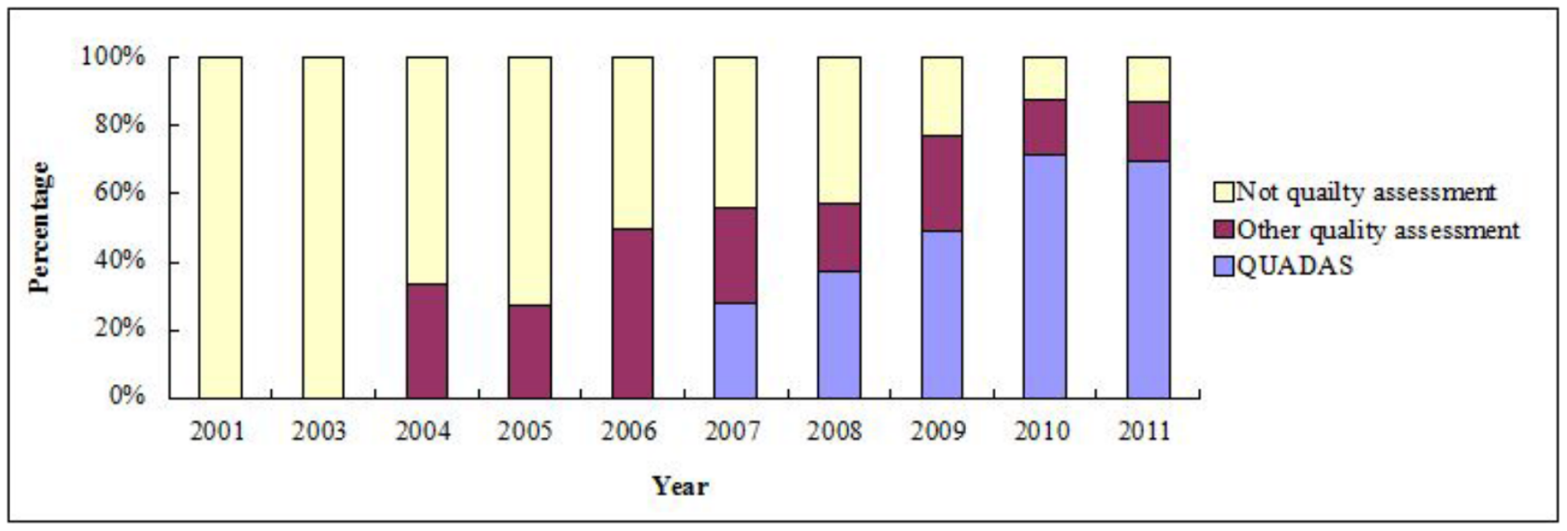
Changing pattern of quality assessment in diagnostic systematic reviews/Meta-analyses.

All reviews published before 2003 reported no formal quality assessment of the primary studies. However, most of (69.6%) reviews used the QUADAS tool for quality assessment in 2011, although other methods of quality assessment were still being used (see Figure 3).

### PRISMA Checklist Assessment (Table 3)

Generally compliance with PRISMA was poor: none of included 312 SRs/MAs fulfilled all 27 items of PRISMA. 70.0% of PRISMA criteria were complied within the following items: title (item 1, 82.4%), rational (item 3, 89.7%), eligibility criteria (item 6, 88.1%), information sources (item 7, 87.8%), summary measures (item 13, 74.4%), synthesis of results (item 14, 85.3%), study characteristics (item 18, 78.8%), results of individual studies (item 20, 84%), summary of evidence (item 24, 85.6%). Total compliance with PRISMA items was less than 30.0% in structured summary (item 2, 22.4%), objective (item 4, 18.9%), protocol and registration (item 5, 2.6%), risk of bias across studies (item 15, 26.3%), funding (item 27, 28.8%). Detailed information was presented in Table 3.

**Table 3:** The results of quality assessment of reporting (n = 312).

PRISMA Items		Yes		Partial		No	
		n(%)	95%CI	n(%)	95%CI	n(%)	95%CI
**Title**	**1.Title**	257(82.4)	77.7–86.2	0	–	55(17.6)	13.8–22.3
**Abstract**	**2.Structured summary**	70(22.4)	18.1–27.4	228(73.1)	67.9–77.7	14(4.5)	2.7–7.4
**Introduction**	**3.Rational**	280(89.7)	85.9–92.7	0	–	32(10.3)	7.1–14.1
	**4.Objective**	59(18.9)	14.9–23.6	247(79.2)	74.3–83.3	6(1.9)	0.9–4.2
**Methods**	**5.Protocol and registration**	8(2.6)	1.3–5.0	0	–	304(97.4)	95.0–98.7
	**6.Eligibility criteria**	275(88.1)	84.1–91.3	19(6.1)	3.9–9.3	16(5.1)	3.2–8.2
	**7.Information sources**	274(87.8)	83.7–91.0	0	–	38(12.2)	9.0–16.3
	**8.Search**	120(38.5)	33.2–44.0	129(41.3)	36.0–46.9	63(20.2)	16.1–25.0
	**9.Study selection**	134(42.9)	37.6–48.5	62(19.9)	15.8–24.7	116(37.2)	32.0–42.7
	**10.Data collection process**	193(61.9)	56.3–67.1	40(12.8)	9.5–17.0	79(25.3)	20.8–30.4
	**11.Data items**	148(47.4)	41.9–53.0	51(16.3)	12.6–20.9	113(36.2)	31.1–41.7
	**12.Risk of bias in individual studies**	204(65.4)	59.9–70.5	13(4.2)	2.4–7.0	95(30.4)	25.6–35.8
	**13.Summary measures**	232(74.4)	69.2–78.9	0	–	80(25.6)	21.1–30.8
	**14.Synthesis of results**	266(85.3)	80.9–88.8	28(9.0)	6.3–12.7	18(5.8)	3.7–9.0
	**15.Risk of bias across studies**	82(26.3)	21.7–31.4	4(1.3)	0.5–3.4	226(72.4)	67.2–77.1
	**16.Additional analyses**	122(39.1)	33.8–44.6	8(2.6)	1.3–5.0	185(59.3)	53.8–64.6
**Results**	**17.Study selection**	141(45.2)	39.8–50.8	82(26.3)	21.7–31.4	89(28.5)	23.8–33.8
	**18.Study characteristics**	246(78.8)	74.0–83.0	20(6.4)	4.2–9.7	46(14.7)	11.2–19.1
	**19.Risk of bias with studies**	201(64.4)	59.0–69.5	12(3.8)	2.2–6.6	99(31.7)	26.8–37.1
	**20.Results of individual studies**	251(80.4)	75.7–84.5	23(7.4)	4.9–10.8	38(12.2)	9.0–16.3
	**21.Synthesis of results**	235(75.3)	70.2–79.8	30(9.6)	6.8–13.4	47(15.1)	11.5–19.5
	**22.Risk of bias across studies**	98(31.4)	26.5–36.8	3(1.0)	0.3–2.9	211(67.6)	62.2–72.6
	**23.Additional analyses**	131(42.0)	36.6–47.5	7(2.2)	1.1–4.6	174(55.8)	50.2–61.2
**Discussion**	**24.Summary of evidence**	267(85.6)	81.2–89.1	37(11.9)	8.7–15.9	8(2.6)	1.3–5.0
	**25.Limitations**	212(67.9)	62.6–72.9	40(12.8)	9.5–17.0	60(19.2)	15.2–24.0
	**26.Conclusions**	155(49.7)	44.2–55.2	107(34.3)	29.2–29.7	50(16.0)	12.4–20.5
**Funding**	**27.Funding**	90(28.8)	24.1–34.1	0	–	222(71.2)	65.9–75.9

### Subgroup Analysis on the Quality of Reporting (Figures 4–8)

#### Comparison of the quality of reporting of included SRs/MAs based on the publication time (≤2008 vs. ≥2009)

**Figure 4:**
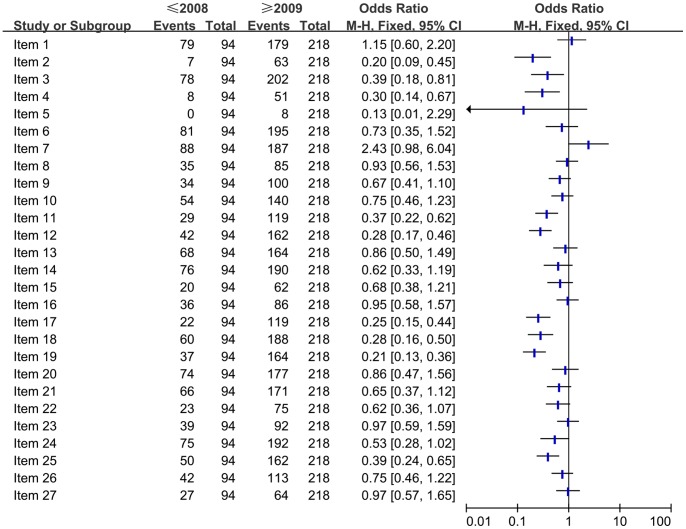
Comparison of the quality of reporting of included systematic reviews/Meta-analyses based on the publication time.

To determine whether there had been an improvement in the quality of reporting after the PRISMA statement was released. The full compliance in each of the PRISMA criteria were compared between the period in or before 2008 and the period in or after 2009 (Figure 4). The results showed that there was an improvement in the following items after the PRISMA statement was released, which was significant difference (*P*<0.050): structured summary (item 2), rational (item 3), objective (item 4), data items (item 11), risk of bias in individual studies (item 12), study selection (item 17), study characteristics (item 18), risk of bias with studies (item 19), limitations (item 25). However, other items didn’t show significant difference or there was no improvement after the PRISMA statement was released.

#### Comparison of the quality of reporting of included SRs/MAs based on the affiliations (Hospital vs. University)

**Figure 5:**
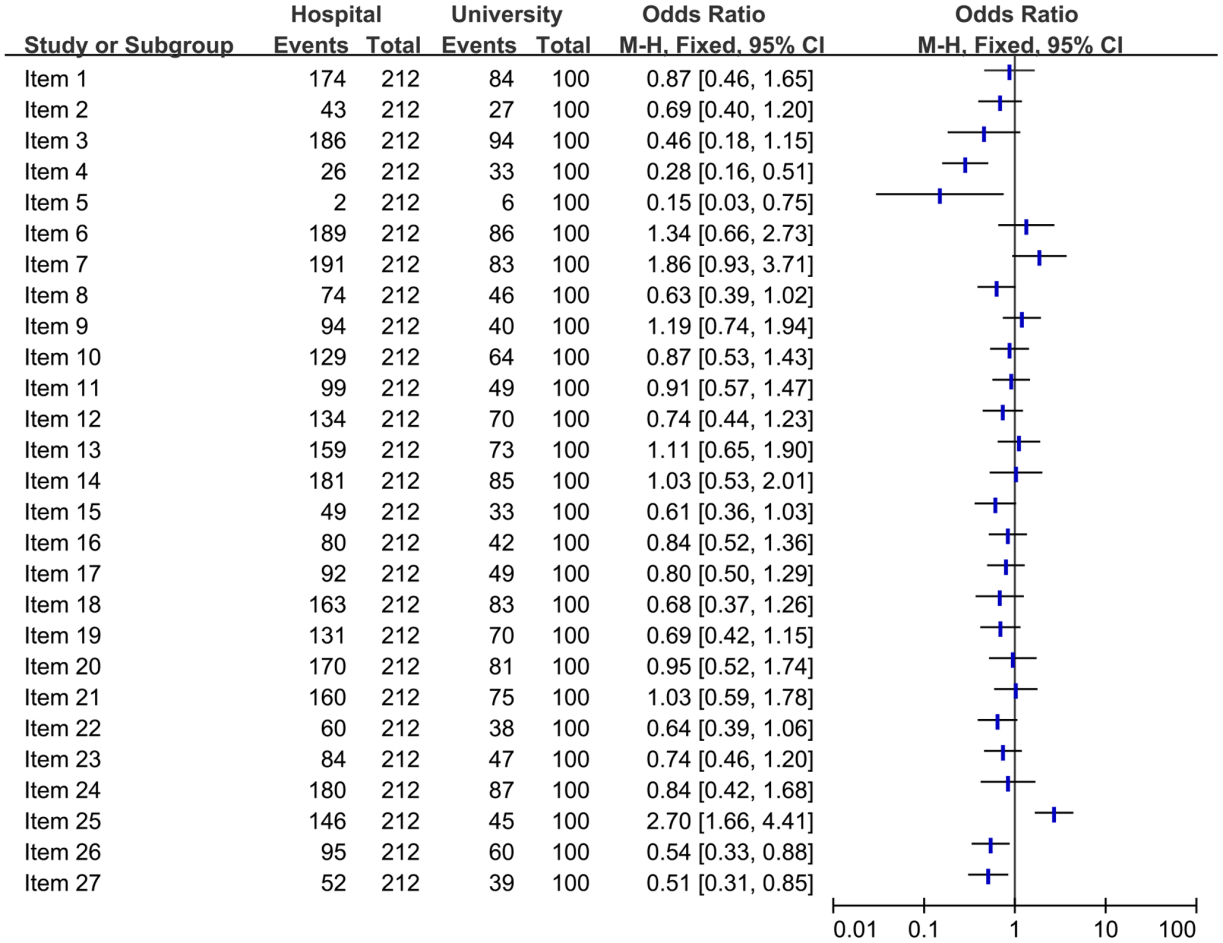
Comparison of the quality of reporting of included systematic reviews/Meta-analyses based on the affiliations.

To investigate whether there had been a difference in the quality of reporting in different affiliations. SRs/MAs published by hospital were compared with that published by university for full compliance in each of the PRISMA criteria (Figure 5). The studies published by hospital had an improvement, and there had been a significant difference in limitations (item 25).

#### Comparison of the quality of reporting of included SRs/MAs based on the funding sources (Funded vs Non-funded)

**Figure 6:**
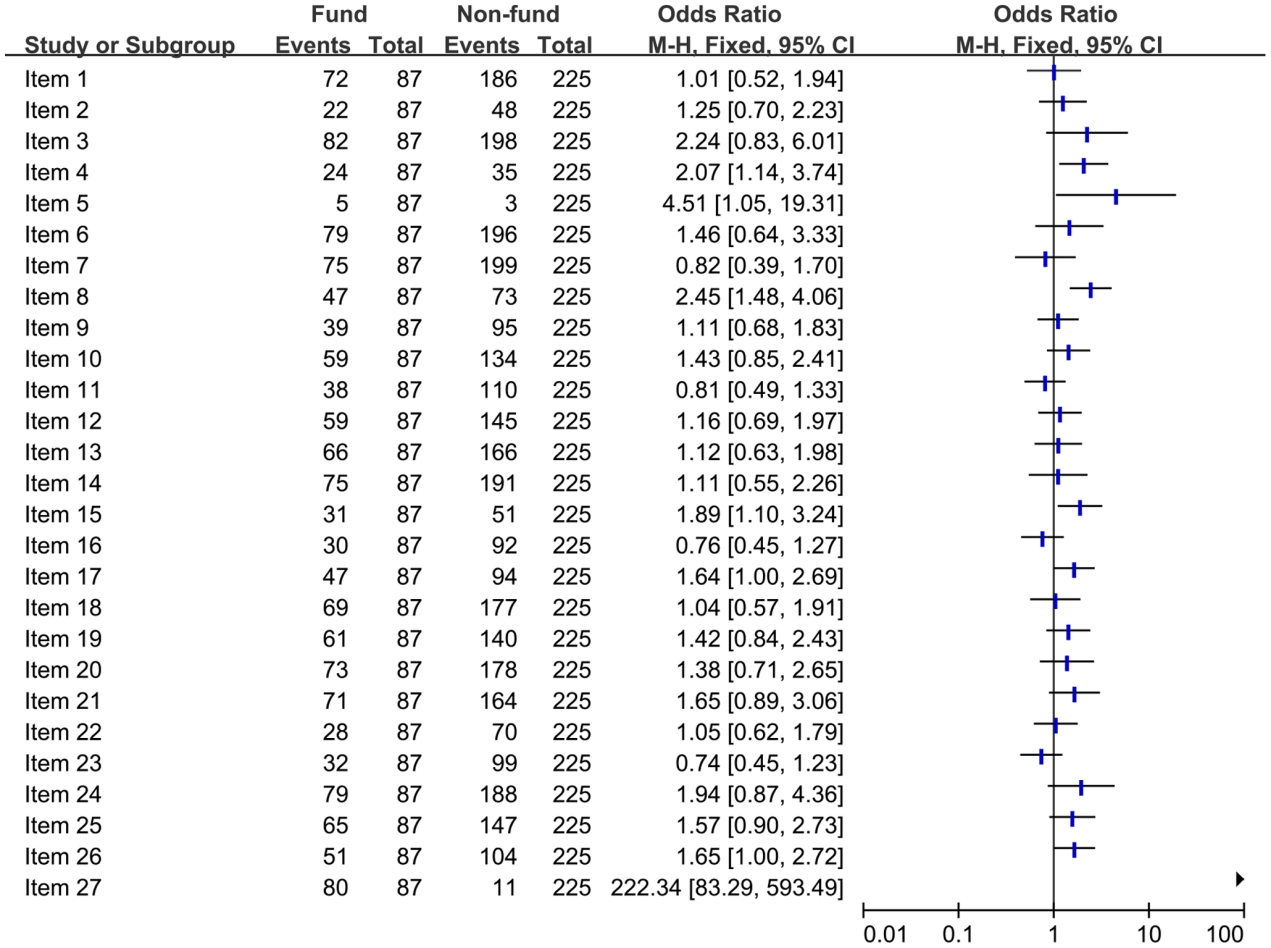
Comparison of the quality of reporting of included systematic reviews/Meta-analyses based on the funding sources.

The funded articles were compared with non-funded articles for full compliance in all the PRISMA criteria (Figure 6). We found that funded articles had an improvement and there was a significant difference (*P*<0.050) in objective (item 4), protocol and registration (item 5), search (item 8), risk of bias with studies (item 15), study selection (item 17), conclusions (item 26), funding (item 27). However, there was no statistical difference in other items.

#### Comparison of the quality of reporting of included SRs/MAs from CSCD vs non CSCD

**Figure 7:**
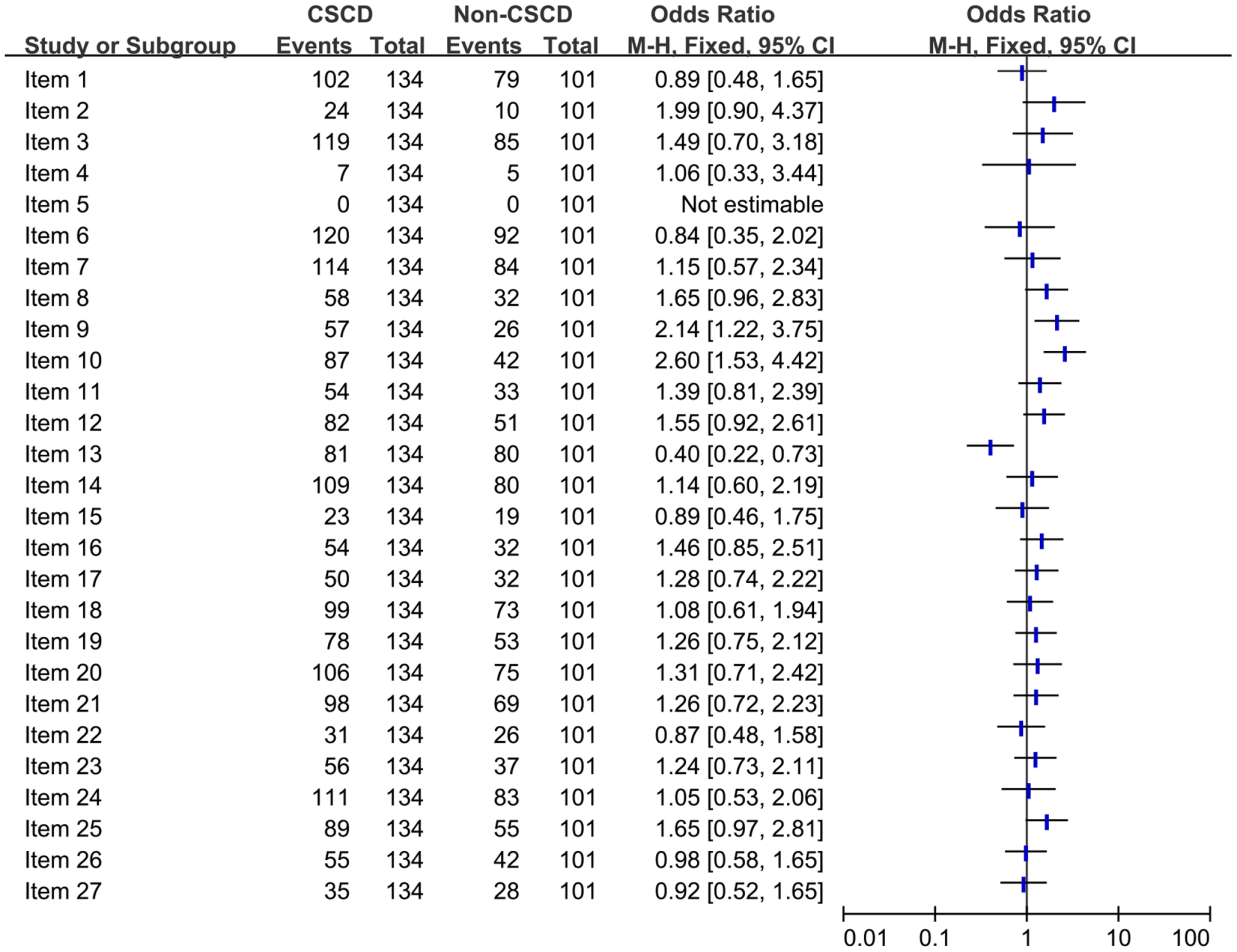
Comparison of the quality of reporting of included systematic reviews/Meta-analyses from CSCD vs non CSCD.

Chinese Science Citation Database (CSCD) has a collection of all core and excellent journals in China. By comparing the quality of reporting of SRs/MAs from CSCD and from non-CSCD, using the PRISMA criteria (Figure 7), we found that there was a statistically significant difference (*P*<0.050) in study selection (item 9), data collection process (item 10), summary measures (item 13). However, there was not statistical difference in other items.

#### Comparison of the quality of reporting of included SRs/MAs from SCI vs non SCI

**Figure 8:**
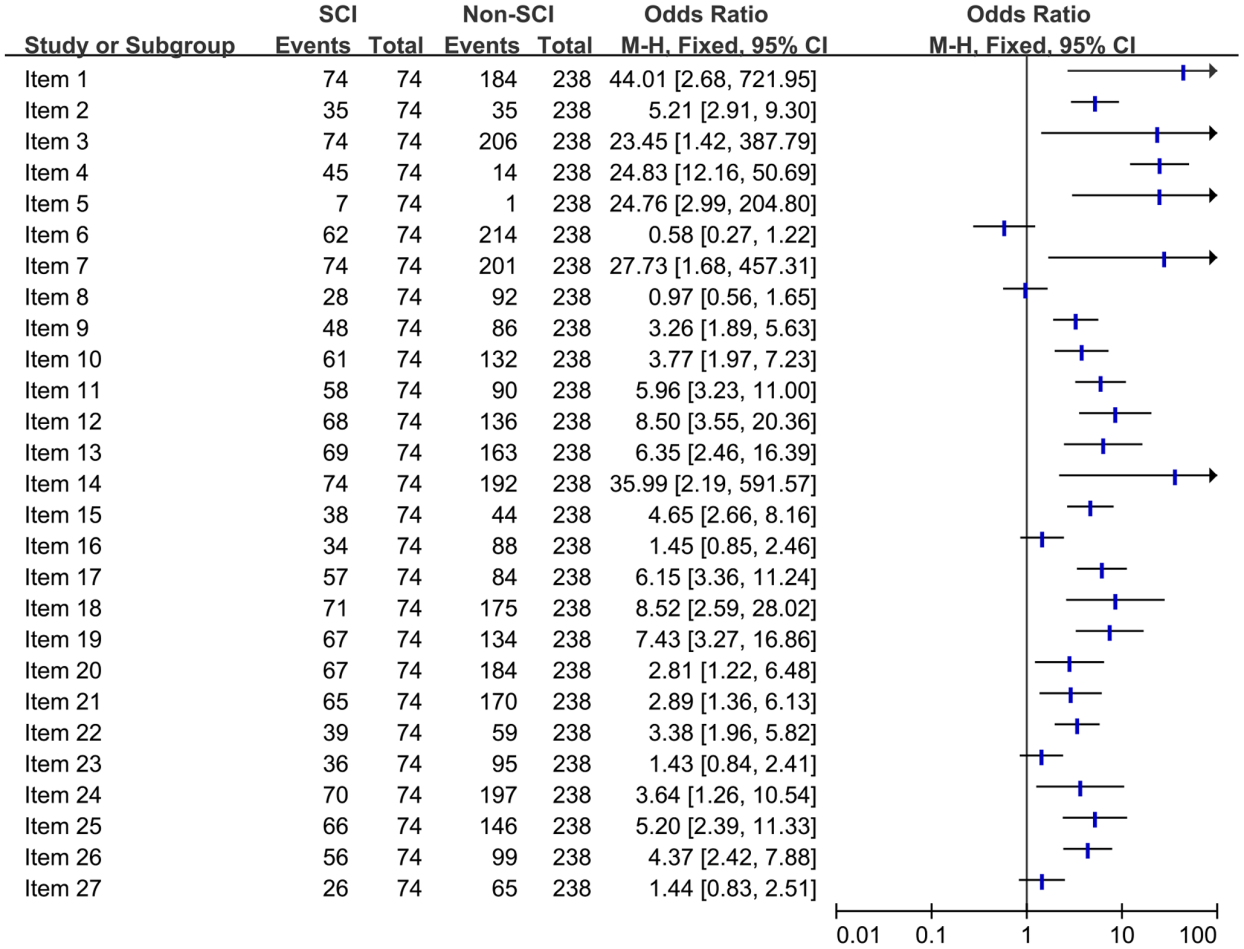
Comparison of the quality of reporting of included systematic reviews/Meta-analyses from SCI vs non SCI.

We included all SRs/MAs published in Chinese and English by authors in China. A comparison was conducted on the quality of reporting between SCI articles and non-SCI articles according to full compliance (Figure 8), there was statistical difference in 22 items (81.5%), and the quality of SCI articles was better compared with non-SCI articles.

## Discussion

### Characteristics of Included Diagnostic SRs or MAs

The number of diagnostic SRs/MAs written by authors in China is increasing annually, 75.3% of SRs/MAs were published in China. Nearly 89.0% were published after the year 2007, a large part of this probably resulted from the introduction QUADAS checklist [14] in 2007. The diagnostic SRs/MAs published by hospitals were 66.3%, indicating that clinicians also devoted to evidence production, which would be beneficial to evidence transforming clinical practice. Only 11.5% research were conducted by 1–2 authors, indicating that the result of data collection and quality assessment were reliable for most of diagnostic SRs/MAs. Most of these articles were published in evidence-based medical journals, and they should be an important evidence sources for diagnostic tests. The impact factor was higher than 5.0 for 12 SRs/MAs. 312 studies involved 15 symptom systems, and neoplasms (42.3%) was a hot topic for researchers. The imaging technologies and laboratory technologies were main diagnostic mode for included studies, which probably due to the fact that it is easy to conform the quantitative index and the testing threshold in imaging technologies and laboratory technologies.

### The Quality of Reporting of Included Diagnostic SRs/MAs

Our PRISMA results showed that the quality of reporting still needs further improvement, the main problems were identified in following four areas: The compliance with PRISMA was not good in structured summary and objectives. The reporting flaws were found in most of (50.0%) included reviews in methods section: protocol and registration, search, study selection, data items, risk of bias across studies, and additional analyses including subgroup analysis and sensitivity analysis). These flaws would affect the quality of diagnostic SRs/MAs. The reporting rate of study selection, risk of bias across studies and additional analyses was less than 50.0% in results section. Even though some of SRs/MAs reported risk of bias across studies and additional analyses in methods section, the analytic results were not presented in results section. Similarly, some of SRs/MAs reported risk of bias across studies and additional analyses in results section, but the analytic results were not reported in methods section. It would affect the integrity and accuracy of researches. The reporting of conclusions was incomplete, most of studies only provided a general interpretation of the results, however, the reporting of implications for future research was poorly provided.

### Comparisons of the Quality of Reporting of SRs/MAs Published by Authors in China and Authors Abroad

Willis BH et al have investigated the quality of reporting of meta-analyses in diagnostic tests published by international authors prior to December 31^st^, 2008 [1]. We compared the total compliance (Yes) in each item of PRISMA by t-test in regarding to SRs/MAs published by authors in China and authors abroad (Text S2). The results showed that the quality of reporting of diagnostic SRs/MAs published by authors in China had more serious reporting flaws in the following items when compared with SRs/MAs published by authors abroad, and the difference was statistically significant (the compliance for PRISMA was less than 50.0%): structured summary (22.4% vs. 34.8%, P = 0.002), objectives (18.9% vs. 66%, P<0.001), risk of bias across studies (methods section, 26.3% vs. 64.4%, P<0.001), risk of bias across studies (results section, 34.4% vs. 57.6%, P<0.001), funding (28.9% vs. 48.3%, P<0.001). It is worth mentioning that the reporting quality of diagnostic SRs/MAs published by authors in China was higher statistically in following items when compared with SRs/MAs published by authors abroad: search (methods section, 38.5% vs. 25.0%, P<0.001), risk of bias in individual studies (methods section, 65.4% vs. 43.6%, P<0.001), study characteristics (results section, 78.9% vs. 55.9%, P<0.001), results of individual studies (results section, 85.0% vs. 68.6%, P = 0.020).

### Limitations

Although both Chinese and English databases were searched, and study selection, data extraction and quality assessment were conducted independently by two reviewers, there are still some limitations to our study due to the different level of understanding PRISMA statement between different researchers. Second, our study only included diagnostic SRs/MAs, however, the SRs/MAs on pathogenesis, prognosis, genetic polymorphism, and intervention were not included. Third, in our search, we searched the term “systematic review” and “meta-analysis” in titles, abstracts or keywords. Some potentially eligible systematic reviews may, however, not use these terms in their titles or abstracts. PRISMA statement is generic checklist aimed at improving the reporting of all types of SRs and does not contain some of the more specific nuances of diagnostic test reviews. However, most of items of PRISMA are applicative regarding to diagnostic SRs/MAs, especially eligibility criteria, search, risk of bias with studies, risk of bias across studies, and additional analysis etc. The quality of reporting for these items was presented in Table 2. Finally, the included reviews evaluated a range of diagnostic tests involving 15 symptom systems and different diagnostic technologies. It is unlikely that the quality of reporting will be completely independent of variation in these factors, therefore, the effect of heterogeneity on the results needs to be considered.

## Conclusion

We have investigated the quality of reporting of 312 SRs/MAs related to diagnostic tests published by authors in China. The results demonstrate that the number of diagnostic SRs/MAs is increasing annually. The quality of reporting has measurably improved over the previous years. Unfortunately, there are still many flaws in the areas including protocol and registration, search, risk of bias across studies, additional analyses. We propose following strategies for future research: Since a protocol can pre-specify the objectives and methods of the systematic review [11], it is important to provide registration information including the registration number. There is great demand to establish a registry platform for SRs/MAs in China. The instructions to authors should include related reporting items, and the submission of PRISMA checklist. The researchers should update timely knowledge on SRs/MAs. The reviewers writing SRs/MAs, especially Chinese reviewers, should pay more attention to area such as search, risk of bias across studies, additional analyses.

## Supporting Information

Text S1 **The detailed search algorithms for each database.**
(DOC)

Text S2 **Comparisons of the quality of reporting of Systematic reviews/Meta-analyses published between authors in China and authors abroad.**
(DOC)

Checklist S1 (DOC)
