# Supplementary material for: The Assessment of the Quality of Reporting of Systematic Reviews/Meta-Analyses in Diagnostic Tests Published by Authors in China
Source: PLoS One. 2014 Jan 21;9(1):e85908. doi: 10.1371/journal.pone.0085908 (PMC3897563; doi:10.1371/journal.pone.0085908)
Supplement: Text S1 — The detailed search algorithms for each database. (DOC) [file pone.0085908.s001.doc]

The syntax for PubMed searches was as follows:

#1 "sensitivity and specificity"[MeSH Terms]

#2 "sensitivity"[All Fields] AND "specificity"[All Fields]

#3 "sensitivity and specificity"[All Fields]

#4 #1 OR #2 OR #3

#5 systematic review* OR meta-analysis*[Title/Abstract]

#6 systematic review[sb] OR meta-analysis[sb]

#7 #5 OR #6

#8 China OR Chinese[All Fields]

#9 #4 AND #7 AND #8

The syntax for EMBASE searches was as follows:

#1 'sensitivity and specificity':ab,ti

#2 'sensitivity' AND 'specificity':ab,ti

#3 #2 OR #3

#4 'meta-analysis':ab,ti

#5 "systematic reviews":ab,ti

#6 "systematic review":ab,ti

#7 "meta analyses":ab,ti

#8 "meta analysis":ab,ti

#9 "meta-analyses":ab,ti

#10 #4 OR #5 OR #6 OR #7 OR #8 OR #9

#11 'China' OR 'Chinese'

#12 #3 AND #10 AND #11

The syntax for The Cochrane Library searches was as follows:

#1 'sensitivity and specificity':ti,ab,kw

#2 'sensitivity' AND 'specificity':ti,ab,kw

#3 #2 OR #3

#4 'meta-analysis':ti,ab,kw

#5 "systematic reviews":ti,ab,kw

#6 "systematic review":ti,ab,kw

#7 "meta analyses":ti,ab,kw

#8 "meta analysis":ti,ab,kw

#9 "meta-analyses":ti,ab,kw

#10 #4 OR #5 OR #6 OR #7 OR #8 OR #9

#11 China OR Chinese

#12 #3 AND #10 AND #11

The syntax for Web of science searches was as follows:

Topic=(systematic review* OR meta analysis*) AND Topic=(sensitivity OR specificity) AND Topic=(China OR Chinese)
