# Supplementary material for: The Assessment of the Quality of Reporting of Systematic Reviews/Meta-Analyses in Diagnostic Tests Published by Authors in China
Source: PLoS One. 2014 Jan 21;9(1):e85908. doi: 10.1371/journal.pone.0085908 (PMC3897563; doi:10.1371/journal.pone.0085908)
Supplement: Text S2 — Comparisons of the quality of reporting of Systematic reviews/Meta-analyses published between authors in China and authors abroad. (DOC) [file pone.0085908.s002.doc]

**Appendix 3**

**Table 2 Comparisons of the quality of reporting of SRs/MAs published between authors in China and authors abroad**

| **PRISMA Items (Yes)** | | **Current study (n=312)** | |  | **Willis' study (n=236)** | | **P-value** |
| --- | --- | --- | --- | --- | --- | --- | --- |
|  |  | **n** | **%** |  | **n** | **%** |  |
| **Title** | **1.Title** | 257 | 82.37% |  | 221 | 93.64% | 0.0002 |
| **Abstract** | **2.Structured summary** | 70 | 22.44% |  | 82 | 34.75% | 0.002 |
| **Introduction** | **3.Rational** | 280 | 89.74% |  | 233 | 98.73% | 0.0003 |
|  | **4.Objective** | 59 | 18.91% |  | 143 | 60.59% | <0.00001 |
| **Methods** | **5.Protocol and registration** | 8 | 2.56% |  | 2 | 0.85% | 0.16 |
|  | **6.Eligibility criteria** | 275 | 88.14% |  | 209 | 88.56% | 0.88 |
|  | **7.Information sources** | 274 | 87.82% |  | 235 | 99.58% | 0.0006 |
|  | **8.Search** | 120 | 38.46% |  | 59 | 25.00% | 0.0009 |
|  | **9.Study selection** | 134 | 42.95% |  | 134 | 56.78% | 0.001 |
|  | **10.Data collection process** | 193 | 61.86% |  | 149 | 63.14% | 0.76 |
|  | **11.Data items** | 148 | 47.44% |  | 127 | 53.81% | 0.14 |
|  | **12.Risk of bias in individual studies** | 204 | 65.38% |  | 103 | 43.64% | <0.00001 |
|  | **13.Summary measures** | 232 | 74.36% |  | 235 | 99.58% | <0.00001 |
|  | **14.Synthesis of results** | 266 | 85.26% |  | 234 | 99.15% | <0.00001 |
|  | **15.Risk of bias across studies** | 82 | 26.28% |  | 152 | 64.41% | <0.00001 |
|  | **16.Additional analyses** | 122 | 39.10% |  | 101 | 42.80% | 0.38 |
| **Results** | **17.Study selection** | 141 | 45.19% |  | 112 | 47.46% | 0.6 |
|  | **18.Study characteristics** | 246 | 78.85% |  | 132 | 55.93% | <0.00001 |
|  | **19.Risk of bias with studies** | 201 | 64.42% |  | 76 | 32.20% | <0.00001 |
|  | **20.Results of individual studies** | 251 | 80.45% |  | 162 | 68.64% | 0.002 |
|  | **21.Synthesis of results** | 235 | 75.32% |  | 235 | 99.58% | <0.00001 |
|  | **22.Risk of bias across studies** | 98 | 31.41% |  | 136 | 57.63% | <0.00001 |
|  | **23.Additional analyses** | 131 | 41.99% |  | 90 | 38.14% | 0.36 |
| **Discussion** | **24.Summary of evidence** | 267 | 85.58% |  | 232 | 98.31% | <0.00001 |
|  | **25.Limitations** | 212 | 67.95% |  | 139 | 58.90% | 0.03 |
|  | **26.Conclusions** | 155 | 49.68% |  | 232 | 98.31% | <0.00001 |
| **Funding** | **27.Funding** | 90 | 28.85% |  | 114 | 48.31% | <0.00001 |
